# Biohybrids for Combined Therapies of Skin Wounds: Agglomerates of Mesenchymal Stem Cells with Gelatin Hydrogel Beads Delivering Phages and Basic Fibroblast Growth Factor

**DOI:** 10.3390/gels10080493

**Published:** 2024-07-25

**Authors:** Farzaneh Moghtader, Yasuhiko Tabata, Erdal Karaöz

**Affiliations:** 1 Nanobiyomedtek Biyomedikal ve Biyoteknoloji Sanayi ve Ticaret Limited Sirketi, Koycegiz 48800, Mugla, Turkey; 2 Laboratory of Biomaterials, Institute for Life and Medical Sciences, Department of Regeneration Science and Engineering, Kyoto University, Kyoto 606-850, Japan; yasuhiko@infront.kyoto-u.ac.jp; 3 Institute of Health Sciences, Stem Cell and Tissue Engineering, Liv Hospital, İstinye University, Esenyurt, İstanbul 34517, Turkey; ekaraoz@hotmail.com

**Keywords:** infected skin wounds, biohybrids, mesenchymal stem cells, gelatin hydrogel beads, bacteriophages (phages), basic fibroblast growth factor

## Abstract

There is great interest in developing effective therapies for the treatment of skin wounds accompanied by deep tissue losses and severe infections. We have attempted to prepare biohybrids formed of agglomerates of mesenchymal stem cells (MSCs) with gelatin hydrogel beads (GEL beads) delivering bacteriophages (phages) as antibacterial agents and/or basic fibroblast growth factor (bFGF) for faster and better healing, providing combined therapies for these types of skin wounds. The gelatin beads were produced through a two-step process using basic and/or acidic gelatins with different isoelectric points. *Escherichia coli* (*E. coli*) and its specific T4 phages were propagated. Phages and/or bFGF were loaded within the GELs and their release rates and modes were obtained. The phage release from the basic GEL beads was quite fast; in contrast, the bFGF release from the acidic GEL beads was sustained, as anticipated. MSCs were isolated from mouse adipose tissues and 2D-cultured. Agglomerates of these MSCs with GEL beads were formed and maturated in 3D cultures, and their time-dependent changes were followed. In these 3D culture experiments, it was observed that the agglomerates with GEL beads were very healthy and the MSCs formed tissue-like structures in 7 days, while the MSC agglomerates were not healthy and shrunk considerably as a result of cell death.

## 1. Introduction

Numerous tissue engineering and regenerative therapy approaches for the restoration of deep skin wounds have been proposed and studied using various skin substitutes [1,2,3]. Open wounds are prone to bacterial invasions, which may result in severe infections in the wound and can even cause systemic infections, making wound healing more difficult and much less successful. Innovative bioengineered skin substitutes and combined regenerative therapy options are needed to reduce mortality and morbidity, in order to save patients’ lives and improve their quality of life.

Bacterial infections are among the most significant health concerns worldwide. Antibiotics have changed medical practice through significantly decreasing the morbidity and mortality associated with bacterial infections. In general, antibiotics have been successfully applied to fight against pathogens since the discovery of penicillin by Alexander Fleming in 1928; however, now we are faced with limitations due to the emergence of antibiotic-resistant bacteria, which is a public health challenge with extensive health, economic, and societal implications [4,5,6,7]. It is clear that there is an urgent need for alternative medicines and pharmaceutical formulations to fight these pathogens.

Phages are typical viruses, which use pathogenic bacteria as hosts to propagate. They recognize their target bacteria very specifically, even at the strain level, deliver their genetic materials into the bacterial cells via their membranes, and let the host reproduce them. A high number of virions are formed in the bacterial cells, and the lytic phages destroy the host bacteria and the virions emerge, which are ready to attack target bacteria in the surrounding area. The whole reproduction process takes less than an hour. Virions are self-expanding entities but also self-limiting, which means that they control the pathogenic bacterial population quite successfully; therefore, they are known as natural antibacterial agents. Phages are known to be harmless to humans. These astonishing properties and advantages over traditional antibiotics have been reported in the relevant literature [8,9,10,11,12,13,14,15,16]. Phages were discovered and described in the first quarter of the 20th century. Several phages, used mainly as cocktails, have already been developed, commercialized, and applied clinically in Eastern Europe (mainly Russia and Georgia) for a long time. During this period, the Western world was using antibiotics. However, as mentioned above, there are too many bacterial strains with antibiotic resistance at present, and a promising way to remedy this situation is the use of phages.

bFGF is one of the most important members of the FGF family, which plays several key roles in wound healing [17,18,19,20,21,22,23]. In 1988, Kaken Pharmaceutical Co., Ltd., Tokyo, Japan, licensed the rights for a recombinant human bFGF, which was originally developed for patients with skin ulcers. Several formulations have already been used over the past two decades at all levels and with very successful clinical outcomes, mainly in Japan [22].

Gelatin, a natural biopolymer (i.e., a polypeptide) derived from collagen, has gained significant attention in the field of biomaterials due to its unique properties. It has very high biocompatibility, it is biodegradable with bioactive degradation products (peptides) that trigger tissue healing, and gelatin hydrogel matrices can be shaped under mild conditions (i.e., at room temperature and without the use of potentially harmful chemicals), which is particularly important for loading environmentally sensitive bioactive agents into controlled release systems without loss of their activity. Therefore, gelatin has become one of the preferred choices as a biomaterial in diverse applications [23,24,25,26].

In recent years, stem cells have replaced primary cells in cell therapies and tissue engineering. Multipotent mesenchymal stem cells have a high potential for clinical applications; they can be isolated from various tissues, such as bone marrow, adipose tissue, the umbilical cord, and the placenta, and they can be easily expanded, differentiated into multiple cell types, evade the immune system, and possess immune-suppressive properties [27,28,29]. The cells (including stem cells) used in regenerative medicine and tissue engineering are commonly prepared in 2D cultures; however, there is a more challenging alternative: using 3D cultures in which cells interact with each other and the extracellular matrix, similar to in vivo conditions, resulting in a much higher chance of proper and rapid tissue formation. Multicellular spherical cell clusters are also referred to as spheroids and have received significant attention. Various alternative methods have been described and reported in the relevant literature for the formation of cell clusters and their application, either alone or as biocomposites with tissue scaffolds [27,28,29,30,31].

In this study, stem cell clusters were also formed, but they are not strictly spheroids composed of only cells. Instead, they are 3D cell agglomerates of MSCs with GEL beads. It was anticipated that the cell clusters would be connected and matured into tissue-like structures. The presence of GEL beads enables a high mass transfer within the agglomerates; therefore, cell death even in the central parts could be significantly reduced, compared to the high level of cell death in the cores of spheroids [30,31,32]. It is further expected that the GEL beads will gradually biodegrade in vivo, resulting in degradation products, such as peptides, which will positively influence cell viability and the further maturation of the newly forming tissue-like structures.

This article describes the preparation of bioactive GEL beads carrying and controlled releasing T4 phages and bFGF and the formation of MSC agglomerates with or without GEL beads, as well as providing a comparison of in vitro 3D cell cultures with these alternative biohybrids.

## 2. Results and Discussion

### 2.1. Propagation of the Target Bacteria (E. coli) and Its T4 Phages

*Escherichia coli* (*E. coli*) was used both as the host for propagation of the T4 phages and also as the target bacteria for the phage activity and performance tests. The *E. coli* strain used here was obtained from ATCC^®^ (Manassas, VA, USA) and cultured within LB medium. In the bacterial culture medium, there was a time lag of about 1 h, after which steady-state growth was achieved. The applied optical density (OD_600_) of 0.5 corresponds roughly to a bacterial concentration of 10^8^ CFU/mL, which was considered to be very suitable for the propagation of T4 phage emulsions with high concentrations for further studies.

**Figure 1:**
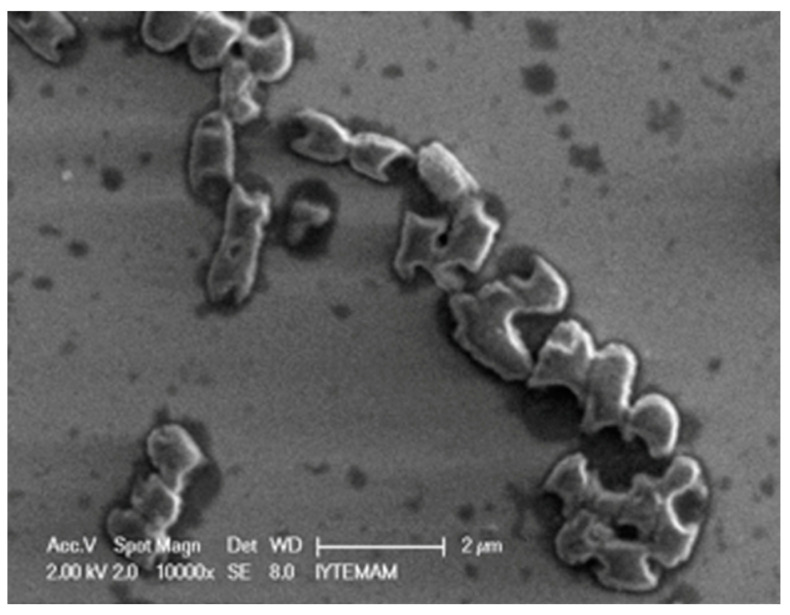
Representative image demonstrating interaction of *E. coli* with its T4 phages on the SEM substrate surface.

A representative Scanning Electron Microscope (SEM) image demonstrating the interaction of the target bacteria *E. coli* with its specific T4 phages (those propagated in this study) on the SEM substrate is shown in Figure 1. This image was captured just after dropping the T4 phage emulsion on the top of the *E. coli* layers on the substrate surface. The phages can be seen to have recognized their target bacteria quite selectively and started to destroy their targets. This is an early-stage image and, therefore, most bacterial cells are intact; however, some of them were already being attacked. The phages were able to destroy their targets very quickly, with almost no intact bacteria left and the surface presenting an oily appearance within less than an hour [33].

### 2.2. Production of Gelatin-Based Hydrogel Microbeads

In this study, the GEL beads were prepared through a two-step process. In the first step, gelatin spherical gels were formed. Note that the average size and the size distribution of the gelatin beads can be adjusted by changing several parameters related to the recipe and preparation conditions, including the gelatin source, type, and molecular weight, the aqueous phase composition and other properties, the dispersion phase type, composition, and properties, the aqueous phase/dispersion phase ratio, the agitation type, rate, and duration, and the temperature. We did not attempt to optimize either the recipe or processing parameters in this study; instead, we followed the earlier work of Tabat et al. when considering our preliminary studies, and accordingly selected the recipe and conditions to produce the GEL beads with proper sizes (around 50 µm) to be used for the formation of MSC agglomerates with the desired sizes and structures [18,32,34].

In the second step, the uncross-linked gelatin gel beads were dried and physically cross-linked via dehydrothermal treatment. Note that the cross-linking density can be easily controlled by changing the treatment time [18,32,34]. Following our preliminary studies and earlier experiences, we prepared the cross-linked GEL beads with a dehydrothermal treatment time of 24 h. Higher cross-linking densities (less swelling) were observed when we increased the treatment time in our hydrothermal cross-linking process; however, it was noted that changes in both swollen size and swelling extent (water uptake) were observable but not significant (not presented here).

**Figure 2:**
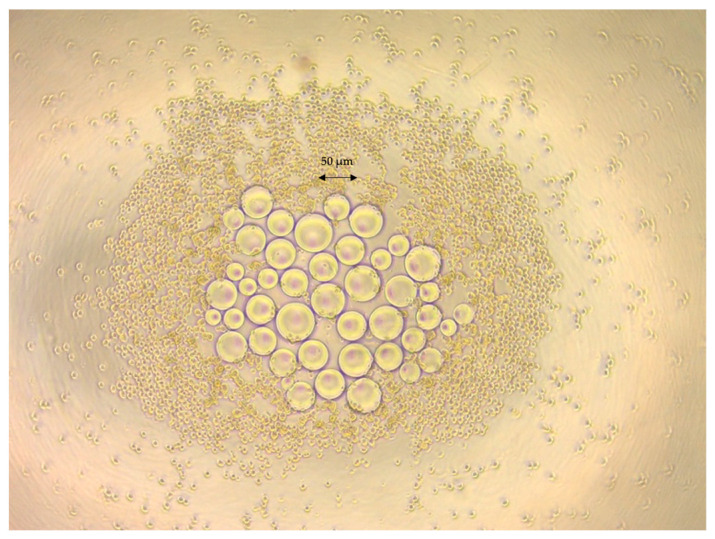
Representative image of the GEL beads and MSCs prepared in this study.

A representative microscopic picture of the swollen GEL beads prepared and used in this study is shown in Figure 2, with the MSCs visible in the same image. The beads can be observed to be quite spherical in shape with quite smooth surfaces. The swelling ratio of the GEL beads was obtained and is presented here in terms of the water content (by weight percentage). The size distributions were as expected, which was the result of the mechanical stirring that was applied in the gelation reactor in the first step; therefore, we fractionated the outcome (as mentioned above) in order to obtain GEL beads with a narrow size distribution. The average diameters and water content percentages (by weight) were 44.5 ± 9.4 μm and 98.1 ± 1.4, respectively.

### 2.3. Phage and bFGF Release from the GEL Beads

**Figure 3:**
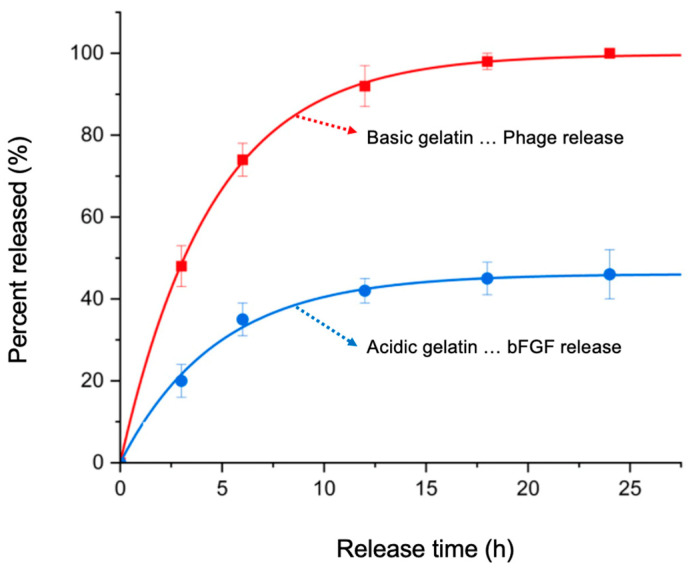
Phage and bFGF release from GEL beads formed of basic and acidic gelatin.

The T4 phages propagated in this study were loaded within GEL beads prepared using native basic gelatin (IEP: 9.0, positively charged at pH 7.4). The phage emulsions, with a concentration of 10^8^ PFU/mL, were loaded into the beads during the swelling process. The loadings were almost 100%, as the aqueous phase volume used was about the volume that the dried GEL beads could absorb (which was determined in the pre-tests). Then, phage release from the GEL beads prepared using basic gelatin (IEP: 9.0) was observed. A representative curve is shown in Figure 3 (the red curve). As expected, there was quite fast phage release: about 75% of the phages loaded were released in 6 h, and release was completed in 24 h. Actually, fast phage release was an intended target of this study, as this would enable quick interventions in cases of infected wounds. It should be noted that, in most cases, open wounds are prone to bacterial attacks in the hospital environment; therefore, speedy actions are required to prevent infections and initiate phage activity events as early as possible. This means that the developed phage-carrying GEL beads can be considered as suitable formulations to reach this target.

bFGF is a very important growth factor which plays important roles in many bioprocesses (e.g., angiogenesis) involving tissue regeneration [17,18,19,20,21,22]. However, when it is used in free form in vivo, it may lose its activity in a very short time. Therefore, the most suitable strategy to maintain its activity for longer periods during in vivo use is its sustained release from a carrier matrix, such as the GEL beads prepared in this study. The IEP of bFGF is 9.6, which means that it is positively charged at pH 7.4. Therefore, basic gelatin—which is also positively charged—is not a good choice for the preparation of GEL beads, as it enables the rapid release of bFGF (not presented here). Therefore, we used GEL beads prepared from acidic gelatin, which is negatively charged at pH 7.4, as suggested in the related literature [18,33,34]. As expected, the sustained release of bFGF from these GEL beads was observed, as exemplified in Figure 3 (the blue curve).

### 2.4. Agglomerates of MSCs with or without GEL Beads

Mesenchymal Stem Cells (MSCs) were isolated from adipose tissue of mice. The isolated cells were 2D-cultured in αMEM media. These culture studies lasted for about 7–14 days, in order to reach cell densities of 1 × 10^4^ cells/cm^2^ and above in the petri dishes. The MSCs isolated from adipose tissue, cultured, and used for the preparation of the biohybrid agglomerates exhibited a typical fibroblastic cell morphology around the GEL beads, as demonstrated in Figure 2.

For the preparation of the 3D clusters, multi-well, round-bottom cell culture plates were coated with PVA to make the surfaces hydrophilic, thus preventing cell adhesion and enabling the clustering of the MSCs—alone or in combination with the GEL beads. Tabata et al. previously prepared similar clusters using different types of cells (both primary cells and cell lines) and varying amounts and sizes of gelatin beads [32,34]. Note that the GEL beads used in this group of experiments were prepared from acidic natural gelatin (IEP: 5.0). In the scope of this study, initially, each well of the culture plate was filled with 50–100 µL of 1 × 10^3^ cells/mL MSC suspension or 50–100 µL of 1 × 10^3^ cells/mL MSC suspension + 50–100 µL of 1 × 10^4^ beads/mL gelatin suspension, then incubated for 7 days. It should be noted that one of the parameters undoubtedly affecting the size and performance of biohybrid structures is the numerical ratio of the MSCs to GEL beads used initially. This ratio not only affects the size of the resulting clusters, but also their performance. Tabata et al. extensively studied the effects of this parameter and obtained successful results when starting with a numerical ratio of 10³/10⁴ cells to GEL beads, which led to the formation of biohybrid clusters with high performance and sufficient physical stability. Considering these observations, clusters were formed in the presented study starting with this ratio.

In their studies, Tabata et al. kept the initial number of beads constant while varying the bead size. As the bead size increased, the agglomerates showed better development, resulting in a higher number of viable cells. However, it should be considered that, as the bead size increases, cell–cell contacts become more challenging. Additionally, maintaining the cluster as a single entity becomes more difficult, and the cluster becomes physically weaker. When planning the experiments in this particular group of the presented study, attention was paid to this aspect, and the swollen GEL bead size was selected to be around 50 μm. As a result, after a 7-day incubation period, the formation of a cell network structure, where cells grew/proliferated and remained connected, ensured the physical integrity of the agglomerates.

**Figure 4:**
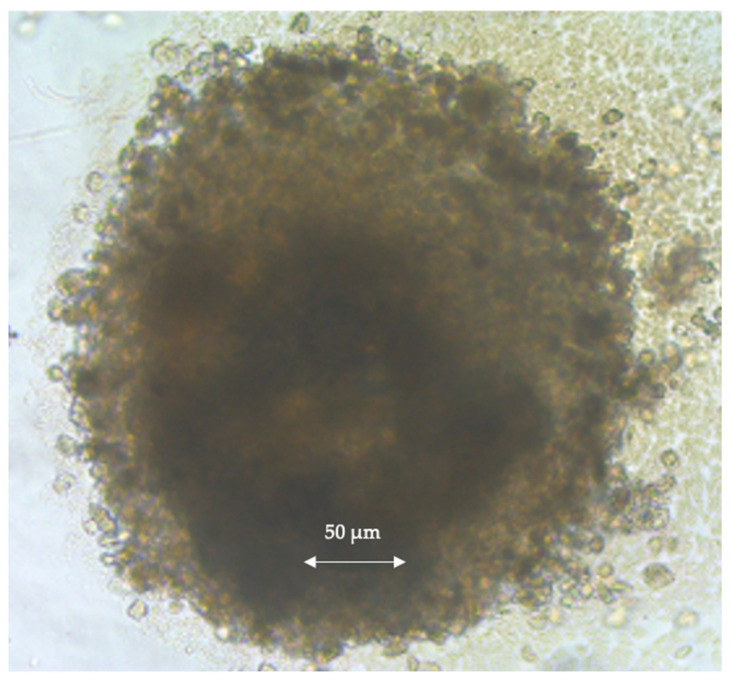
Representative image of the MSCs and GEL agglomerates on the 7th day of 3D culture.

Representative images of the clusters of the MSCs plus the GEL beads taken after the first 24 h and on the 7th day after cluster formation are shown in Figure 4. The round-bottomed culture wells assured spherical cluster formation, as expected. It should be noted that the initial cluster size changed significantly, depending on the cell quantity and the GEL bead size and quantity used (not all presented here).

At the very beginning, the cells and GEL beads were separate, with no fusion, as shown in Figure 2. After 24 h, distinct spherical structures emerged, with cells proliferating and spreading around the GEL beads, resulting in a slight increase in cluster size. On the 7th day, an increase in cell density and integration and a continued increase in cluster size were observed, as exemplified in Figure 4. The clusters formed by MSCs + GEL beads initially had sizes of around 150–200 µm, which increased to approximately 250–300 µm after 7 days of incubation. In contrast, the clusters prepared only from MSCs had sizes of approximately 100–120 µm initially, which decreased to 80–100 µm (or even lower) after 7 days.

The prominent findings and discussions of those earlier studies and the related published data are as follows: It is possible to create 3D clusters from cells alone, without GEL beads; however, as the cell number increases, the agglomerate size also increases and, due to difficulties in the oxygen and nutrient transfer necessary for cell growth (resulting from a simple diffusion barrier), cell death occurs in the inner regions of the clusters. This phenomenon is observed in all 3D cell clusters prepared using different methods, including spheroids [27,28,29,35,36]. Cell death in the inner regions of 3D structures is inevitable and remains a significant issue that needs to be addressed. In the scope of the presented study, although a relatively low number of cells were used to form the agglomerates, cell death was still noticeable. Undoubtedly, reducing the size of the clusters can decrease cell death due to this issue. However, as cell growth initiates, the size will increase again, re-introducing the problem. Therefore, reducing the cell number should not be considered as a viable solution.

### 2.5. Cell Counts and Metabolic Activities

**Figure 5:**
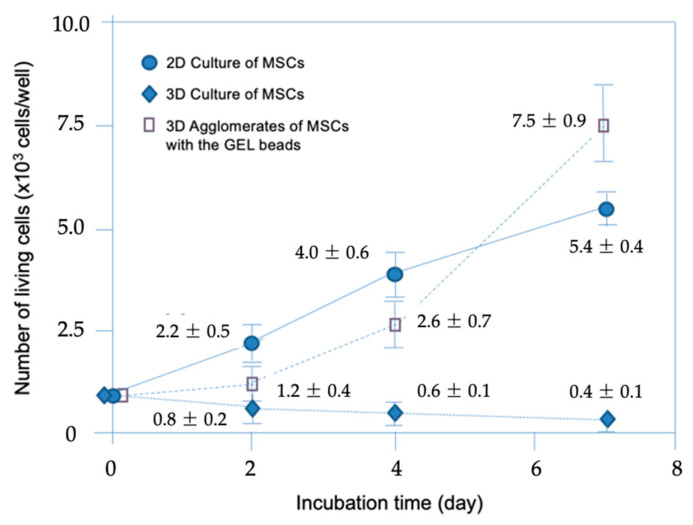
Changes of the living cell counts in 2D and 3D cultures.

The time-dependent changes in the live cell counts in the MSC clusters (with and without GEL beads) and MSCs throughout the 7-day 3D and 2D cultures were determined. The mean values and standard deviations (obtained from data collected in at least three repeated experiments) are presented in Figure 5. It can be observed that the viability of the clusters prepared solely from MSCs decreased over time, dropping below half of the initial value within 7 days. In the first 4 days, the live cell count increased in the 2D monolayer culture. However, in the biohybrid 3D agglomerates, the live cell counts reached higher values (7.5 × 10^3^) after 7 days, compared to the 2D culture. The cell viability in MSCs + the GEL beads was similar to that observed in 2D monolayer cultures and, even after a 7-day incubation period, starting with the same cell number, the number of viable cells reached in the 3D culture in the MSCs + GEL beads group was significantly higher than that in the 2D culture. However, in MSC clusters alone (without gelatin beads), the cell death over a 7-day period was significant.

The differences in the changes of the cell counts in the 2D and 3D cultures were statistically significant (*p* < 0.05), as determined by applying one-way analysis of variance with Tukey’s test.

**Figure 6:**
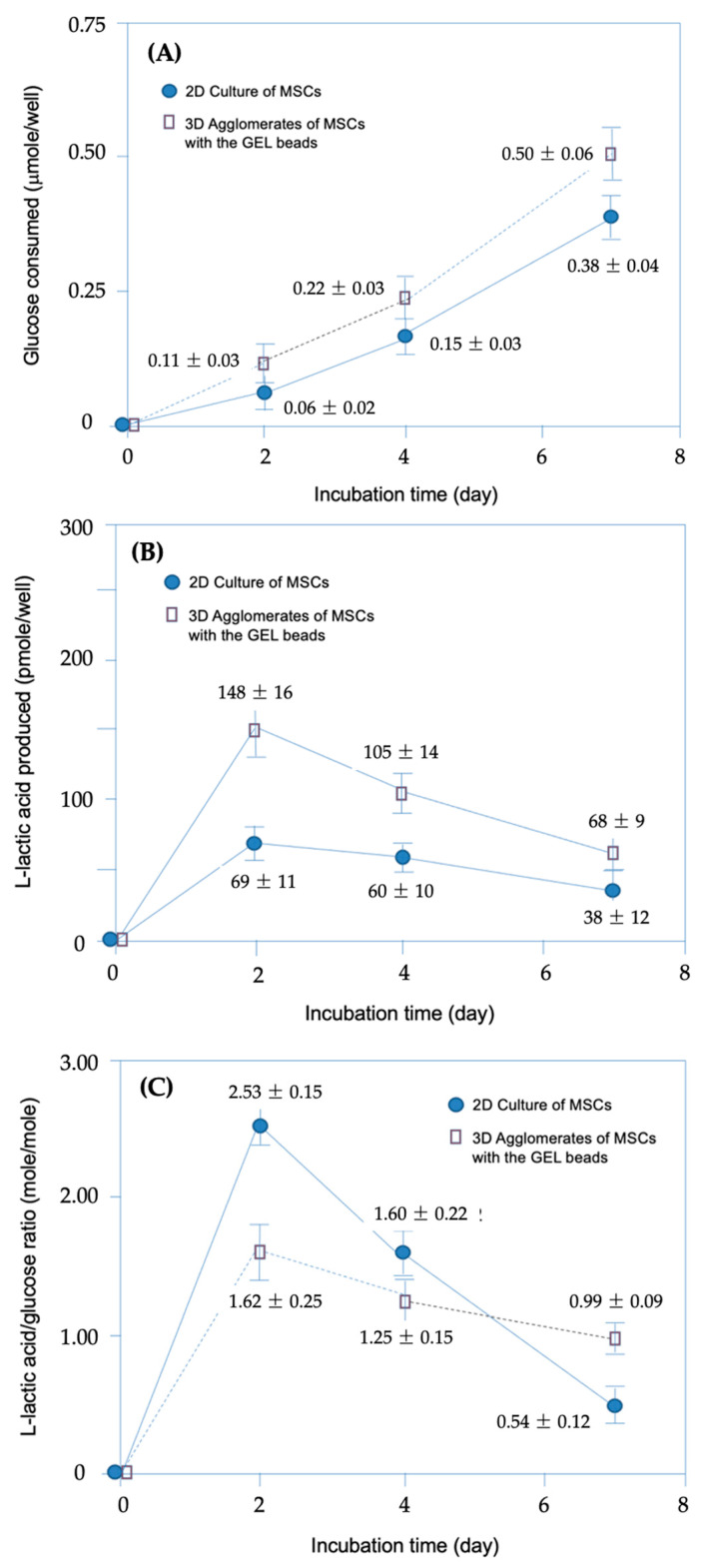
Metabolic activities in 2D and 3D cultures: (**A**) Glucose consumed; (**B**) L-lactic acid produced; and (**C**) changes in the L-lactic acid/glucose ratio.

In the experiments conducted to describe and compare the changes in metabolic activities during the 7-day incubation period in 2D and 3D cultures, glucose consumption and L-lactic acid production were measured, and the L-lactic acid/glucose ratio was evaluated as an indicator of metabolic activity. Figure 6A shows that glucose consumption increased in parallel with the cell count. At the end of the 7-day incubation period, the 3D culture, which exhibited a much higher live cell count, showed significantly higher glucose consumption compared to the 2D culture. Figure 6B presents the time-dependent changes in L-lactic acid production, from which it can be seen that the L-lactic acid synthesis in 2D cultures was lower than that in 3D cultures throughout the incubation period. Figure 6C shows the time-dependent changes in the ratio of L-lactic acid synthesis normalized by the live cell count to glucose consumption. Although the 2D culture exhibited a higher ratio during the first 4 days of culture, the 3D culture showed higher L-lactic acid production per glucose consumption after the 7-day incubation period.

The L-lactic acid production/glucose consumption ratio was evaluated as a metabolic activity indicator, as it has been reported that this ratio serves an indicator of how efficiently the aerobic mechanism is functioning, with a lower ratio indicating a better functioning aerobic mechanism [30,31,37]. Interpreting the data presented here, which compares the results of the 2D monolayer cell cultures with those of the agglomerates of MSCs + GEL beads in 3D culture, the following can be observed: Initially, the aerobic mechanism appeared to work better in the 3D culture despite diffusion limitations, which can be attributed to better cell–cell contacts in the 3D culture. After a 7-day incubation period, cell contacts sufficiently increased in the 2D culture as well, and metabolic activity was higher—especially due to the absence of oxygen diffusion limitations (lower L-lactic acid/glucose ratio). It was expected that diffusion limitations would arise as the cell number increased and the cell network structure became denser. This was manifested in the lower metabolic activity, compared to the 2D culture.

The differences between the changes in metabolic activities (both glucose and L-lactic acid levels) in the 2D and 3D cultures were statistically significant (*p* < 0.05), as determined through one-way analysis of variance with Tukey’s test.

## 3. Conclusions

This study presented 3D biohybrids prepared through the agglomeration of MSCs isolated from mouse adipose tissues and gelatin hydrogel beads. The results demonstrated that these biohybrids can be considered as important potential alternatives to classical tissue-engineered biohybrids composed of biodegradable scaffolds and commonly loaded with primary cells—preferentially stem cells with or without a pre-differentiation step. The forms of these agglomerates are quite different than those of 3D stem cell clusters composed of only cells (i.e., spheroids), as they utilize GEL hydrogel beads, which leads to a functional difference. They are prepared using a very simple technique: adding the dispersions of the cells and GEL beads into vessels coated with PVA, with desired and pre-determined ratios, leading to the self-assembly of clusters. They are then cultured in this 3D format to mature into tissue-like structures, which can be used as biohybrids in regenerative medicine, which was the main focus of the present study.

In addition, the GEL beads in the biohybrids can be considered as bioactive agent delivery vehicles. The loading and controlled release of T4 phages and bFGF, presented in this study, is only an example. Several phages and even antibiotics could be selected and loaded as cocktails to provide a broader antibacterial spectrum, depending on the type and conditions of the disease. Various growth factors and other bioactive molecules could also be loaded, with very high loading yields (even up to 100%), within the GEL beads discussed in this study or different ones with different properties, including different size, charge and cross-linking density, and chemical composition, using not only natural acidic or basic gelatins, but also their chemically modified forms or blends with other biodegradable biopolymers. This would facilitate the development of various targeted release modes and rates for multi-functional effects, enabling diverse uses in combined therapies, even for deep and chronic wounds with severe infections.

## 4. Materials and Methods

### 4.1. Materials

The host and target bacteria—*Escherichia coli K12* (*E. coli K12*), which is the most-studied *E. coli* strain and is considered safe to use in standard laboratories with a Biosafety Level of 1- (BL1)—and its specific T4 phages were obtained from the American Type Culture Collection (ATCC^®^ 11303^TM^ and 11303-B4^TM^, respectively; Manassa, VA, USA) and used as a model couple. Both native basic and acidic gelatins—with isoelectronic point (IEP) of 9.0 and 5.0, respectively—but with the same average molecular weight (of 100 kDa) were obtained from Nitta Gelatin Co. (Osaka, Japan). Basic fibroblast growth factor (bFGF; Sigma-Aldrich, Saint Louis, MO, USA) with a molecular weight and IEP of 16 kDa and 9.6, respectively, was used. An ABCAM conjugation kit (Conjugation Kit—Lightning-Link^®^, ABCAM, ab102884, Cambridge, MA, USA) was used for Fluorescein 5-isocynate (FITC) labeling. For surface hydrophilization, polyvinyl alcohol (PVA) was obtained from Unichika (Tokyo, Japan), with the following properties: polymerization degree of 1800 and a saponification degree of 88% mol. All other chemicals were of reagent grade, obtained also from Sigma-Aldrich (Saint Louis, MO, USA), and were used as received unless noted otherwise.

### 4.2. Propagation of the Target Bacteria and Phages

*E. coli* was propagated using a rather classical protocol, as described previously, in Luria-Bertani (LB) medium [38,39]. Briefly, the host bacterial strain obtained from ATCC^®^ was cultured in the freshly prepared LB medium (25 g of LB in 1 L of distilled water) at 37 °C in a rotary shaker (200 rpm) until reaching the exponential growth phase. The medium was then transferred into 15 mL sterile tubes and centrifuged at 6000 rpm for about 5 min. Then, the obtained pellets were washed few times and re-suspended in phosphate-buffered saline (PBS, pH: 7.2). In order to determine the bacterial concentration, the suspension was diluted with PBS to obtain suspensions with different concentrations. They were then plated/cultured on LB agar (6 g agar in 400 mL of LB media) to estimate the total bacterial (viable) counts (as colony forming units, CFUs), in order to describe the bacterial concentration in the suspension (produced in CFU/mL units) [40].

Phages were propagated using *E. coli* prepared in the previous step as the host, following a rather standard protocol [38,41,42]. Briefly, 100 μL of *E. coli*, freshly prepared with a concentration of 10^8^ CFU/mL and 100 μL of T4 phage (from the stokes) with a concentration of 10^8^ PFU/mL, were mixed and then incubated at room temperature for 15 min, then added to LB medium (supported with CaCl_2_ and MgCl_2_, 0.001 M each). The mixture was incubated for 6 h at 37 °C in a shaking incubator (200 rpm). For purification, the medium was first ultra-filtered through a sterile 0.22 μm filter and then centrifuged at 4 °C and 13,600 g for 20–30 min. The purified phages were re-suspended in sterile PBS buffer (pH: 7.2) or in SM buffer (0.1% gelatin, 100 mM NaCl, 8 mM MgSO_4_, 50 mM Tris-HCl, pH: 7.5) and stored at 4 °C until use. Phage concentration/titers, denoted as “plaque-forming unit per milliliter” (PFU/mL), was determined through a plaque assay technique [32,39,40,41,42]. Briefly, phage emulsions with different concentrations were prepared via dilution of the initial phage suspension. Next, 100 μL from each one and 400 μL of *E. coli* suspension were mixed and added to LB medium (semi-liquid; agar 7.5 g/L) and incubated at 37 °C for 24 h. Then, the lysis plaques were counted.

The performance (activities) of the T4 phages propagated in the previous step—in other words, the abilities of T4 phages to destroy the target bacteria (*E. coli*)—was visualized for bacterial cultures on petri dishes (“soft agar over layers”) [32,39,40,41]. Different volumes of T4 phage nano-emulsions were dropped on the petri dishes carrying *E. coli* cultures on agar, which were then incubated at 37 °C overnight. Note that the prepared *E. coli* lawn plates were originally turbid. However, when *E. coli* were destroyed by the phages, transparent zones were formed due to lysis of the bacteria; these were used to determine the effectiveness of the phages.

SEM micrographs of the target bacteria, its T4 phage, and their interactions were also investigated using a Philips Ultra Plus High Resolution FESEM equipped with an in-lens secondary-electron detector (Philips, Amsterdam, The Netherlands) over an operating range of 2–20 keV, depending on sample charging. The suspensions/emulsions were dropped onto silica slides, dried at room temperature, and the images were then obtained.

### 4.3. Production and Characterization of the Gelatin-Based Hydrogel Microbeads

A two-step protocol was applied to obtain gelatin hydrogel microbeads with different cross-linking degrees and cross-linked network structures, following the protocols described by Tabata et al. [32,34,43]. In the first step, 10 mL gelatin solution (10% *w*/*v*) was heated to 40 °C, then dropped into about 600 mL of a special olive oil dispersion phase (Wako Pure Chemical Industries, Ltd., Osaka, Japan) and dispersed through vortex mixing to yield a water-in-oil dispersion. Gelation was achieved at 4 °C through continuous stirring of the dispersion medium, taking about 1 h to reach gelation. In order to remove the residual olive oil, the gelatin gel microbeads were washed with cold acetone, followed by centrifugation (at 5000 rpm at 4 °C for 5 min), and air-dried. The gelatin hydrogel beads were separated into fractions with different sizes using sieves with different apertures, and the fraction between 32–53 μm was used in the following step. In the second step, the gelatin microbeads were air dried at 4 °C and then dehydrothermally cross-linked to obtain gelatin hydrogel beads in a vacuum incubator at 140 °C under 0.1 Torr vacuum for 24 h.

The swellabilities and water uptake of the cross-linked gelatin hydrogel beads were assessed as follows: The dried hydrogel beads were swollen in distilled water at 37 °C for 24 h to reach the swelling equilibrium. Water uptake was calculated using the weights of the swollen and dried gelatin beads, presented as a percentage. Photographs of gelatin hydrogel beads in the water-swollen state were captured using a microscope (CKX41, Olympus, Tokyo, Japan). In order to calculate the average diameter of the swollen beads, diameters of about 100 hydrogel beads within the sample were measured, and the average values with standard deviations were calculated using the Image J software (NIH, Bethesda, MD, USA) of the microscope.

### 4.4. Phage and bFGF Loading and Release within and from the GEL Beads

For the loading of phages within the gelatin-based hydrogel beads, a very simple protocol was applied. Here, we used T4 phage emulsions with a concentration of 10^8^ PFU/mL. About 200 μL was added into a tube containing 2 mg of cross-linked and dried gelatin-based beads, which was then incubated for 1 h at 37 °C. Note that all the aqueous media was completely taken up by the dried beads, as the volume of the aqueous phase was lower than the water in the wholly swollen beads (which was adjusted in the preliminary studies), meaning that the loading efficiency was almost 100%.

For the phage release experiment, about 200 mg of the freshly prepared GEL beads carrying phages was incubated in 50 mL PBS buffer at pH 7.4 with gentle shaking for up to 24 h. About 100 μL of samples were withdrawn from the medium at selected time intervals (replaced with fresh medium), and the amount of active phages released was assessed using the plaque assay described above. The cumulative amount of phages released during the incubation period was plotted against time, in order to demonstrate the phage release kinetics.

bFGF was first labelled with FITC using the ABCAM conjugation kit, following the protocol described on the company’s website. In brief, 100 µg of FITC was used for 100 µg bFGF. The FITC-labelled bFGF was stored at 4 °C in the dark until use. The GEL beads were loaded with the FITC-labelled bFGF using a very similar loading protocol as that described above for phage loadings. The FITC-labelled bFGF solution containing 100 μg of bFGF (in PBS, pH: 7.4) was taken up into 10 mg dried GEL beads during swelling at 4 °C in the dark for 1 h. Again, the loading efficiency was almost 100%.

bFGF release from the GEL beads was studied in PBS (pH: 7.4) at 37 °C in a shaking incubator with a quite low shaking rate for 48 h. The samples withdrawn from the release medium at selected time intervals were centrifuged, and the fluorescence intensities of the supernatant were measured using a fluorescence spectrometer (Perkin Elmer^®^ Inc., Waltham, MA, USA, ABD). With the fluorescence intensity measurements, the cumulative amount of bFGF released during the incubation period was plotted against time to demonstrate the release kinetics/modes.

### 4.5. Mesenchymal Stem Cells and Their Agglomerates

#### 4.5.1. Isolation and Culture of MSCs

Six female C57BL6/n mice aged 7 weeks (Shimizu Laboratory Supplies, Kyoto, Japan) were used as experimental animals for the Mesenchymal Stem Cells (MSCs) isolation and culture, in accordance with the Animal Ethical Committee of the Baskent University, Medical and Health Sciences Research Council (Ankara, Turkey; approval date, 05/11/2018: No. 18/29). All procedures applied at Kyoto University were also carried out in compliance with the relevant “Animal Ethics Regulations/Guidelines” of Kyoto University, Institute for Life and Medical Sciences (Kyoto, Japan).

The isolation of the Mesenchymal Stem Cells (MSCs) from the adipose tissue of the mice was performed following a method adapted from the related literature [44,45,46], briefly described as follows. Before the isolation process, the experimental animals were anesthetized by applying sevoflurane (Baxter International, Deerfield, IL, USA) as an evaporating anesthetic agent to the desiccator where the animals were placed. The tissues obtained from the subjects were carefully cleaned and washed to remove blood vessels and skin residues, and the following procedures were applied under aseptic conditions in laminar flow cabinets: A mixture of 200 µL of penicillin/streptomycin (Pen-Strep, Sigma-Aldrich, USA) was dissolved in 20 mL of PBS (pH: 7.4) to prepare a 1% Pen-Strep solution. Then, 10 mL of this solution was added to each 10 cm petri dish. The washed tissue pieces were distributed into two petri dishes, and the tissues were roughly minced using a scalpel and scissors. These tissues were transferred to a clean 10 cm petri dish and further minced until a paste-like consistency was achieved. This paste was mixed with a PBS solution containing collagenase. An enzyme solution was prepared by dissolving 50 mg of collagenase enzyme in 20 mL of PBS, followed by sterile filtration through a 0.22 µm filter. The mixture was incubated in a shaking incubator at 37 °C and 120 rpm for approximately 1 h. To stop the enzyme reaction, an equal volume of basal medium (DMEM containing 10% FBS and 1% Pen-Strep) was added to the transferred medium in two centrifuge tubes and centrifuged at 1500 rpm for 10 min to separate the pellet. The following step was performed to lyse red blood cells: The supernatant was carefully aspirated, and the cell pellet was suspended in a 0.83% ammonium chloride (NH_4_Cl; Sigma-Aldrich, USA) solution. Approximately 9 mL of NH_4_Cl solution was used for every 1 mL of pellet. Incubation was carried out at room temperature for 10 min, followed by centrifugation at 1500 rpm for 10 min to sediment the cell pellet. The suspension—which was suspended in approximately 20 mL of culture medium and passed through a 100 µm filter—was sedimented again and transferred to petri dishes with 10 mL of culture medium for cell culturing, and the cell culture described in the following section was applied. The cells that were not used in the short-term were preserved by freezing. For this purpose, the cells, which were centrifuged at 1500 rpm for 10 min at room temperature, were placed into cryogenic vials (Corning Inc., Corning, NY, USA) containing 5–10% dimethyl sulfoxide (DMSO; Sigma-Aldrich, Saint Louis, MO, USA) cooled to 4 °C. The cells were suspended and transferred to liquid nitrogen tanks for storage by placing the vials in containers containing isopropanol (% ≥ 99.5, Sigma-Aldrich, USA) and keeping them at −80 °C overnight.

#### 4.5.2. Formation of Agglomerates of MSCs with or without the GEL Beads

In this group of studies, round-bottomed (U-bottomed) 96- and/or 24-well culture plates were used for the formation of clusters of the MSCs. Initially, these culture plates were coated with hydrophilic polyvinyl alcohol (PVA) to prevent cell adhesion to the well surfaces [30,31,32]. PVA was dissolved in sterile PBS (pH: 7.4) at a concentration of 1% (*w*/*v*), and 100 µL and/or 500 µL of this solution was added to each well (round-bottomed/U-bottomed) of the 96- and/or 24-well culture plates, which were then incubated at approximately 37 °C for 15 min. The solution was aspirated, and the wells were gently washed twice with sterile PBS. For each washing step, 100 µL and/or 500 µL of PBS was used, depending on the well size. In this group of studies, GEL beads were produced using acidic natural gelatin (IEP: 5.0). The swollen GEL beads were washed and sterilized with 70% ethanol using sterilized PBS, which were then used for the formation of cell aggregates, as described below.

To form the biohybrids as agglomerates, the MSCs and GEL beads were placed into bottom-rounded 96-well culture plates pre-coated with PVA. In each well, 50–100 µL of 1 × 10^3^ cells/mL MSCs suspension and 50–100 µL of 1 × 10^4^ particles/mL GEL beads suspension were added slowly, allowing for their agglomeration into spherical masses/clusters. The formation and maturation of the agglomerates were observed in 3D cultures in αMEM culture medium for 7 days. In each well, 1 × 10^3^ MSCs or 1 × 10^3^ MSCs + 1 × 10^4^, the GEL beads were placed, and the cultures were supported with 15% fetal bovine serum (FBS) supplemented with 1% penicillin/streptomycin in αMEM. The cultures were maintained in a CO_2_ incubator at 37 °C, 5% CO_2_, and 95% air with over 90% humidity. Samples were taken from the wells at selected time intervals, and images of the agglomerates were obtained using a special optical microscope (Olympus, Tokyo, Japan) equipped with a camera. The average sizes of the agglomerates, along with their standard deviations, were calculated.

#### 4.5.3. Following the Changes in Cell Counts and Metabolic Activities

The number of living cells in the agglomerates was monitored, for which a protocol based on staining the cell nuclei with crystal violet was applied [30,31,32,36]. During the 7-day culture period, samples were taken from each well of the 24-well cell plates at selected intervals; namely, on the 2nd, 4th, and 7th days. To perform staining, a mixture of 100 µL of 0.2 M citric acid and 0.2% (*w*/*v*) crystal violet was added to each well. After crystal violet staining, a solution of 0.1% (*w*/*v*) Triton X in PBS was added, and the cells were incubated overnight at 37 °C to lyse the cells. The collected nuclei were counted using an optical microscope (Olympus, Tokyo, Japan) and a hemocytometer. It should be noted that the nuclei of living cells are round, while the nuclei of dead cells appear shapeless. Three replicates were performed using three wells in each group, and the mean and standard deviation values were calculated/presented. 

In order to compare cell performances in the 2D and 3D cultures, the metabolic activities with respect to changes in the glucose and L-lactic acid levels were followed in the samples withdrawn from the cell culture media at selected intervals, i.e., on the 2nd, 4th, and 7th days. The glucose levels were obtained using a Glutest Neo Super test kit and a blood glucose meter (glucose dehydrogenase-flavin adenine dinucleotide electrode; Sanwa Kagaku Kenkyusho, Nagoya, Japan). The L-lactic acid levels in the culture medium during the same periods were also determined using an E-lactic acid kit (R-Biopharm AG, Germany). The following protocol, as specified by the manufacturer, was followed: The purchased lyophilized agent was dissolved in water, mixed with the culture medium in a cuvette, and the absorbance was measured at 340 nm. Then, the enzyme solution was added to the cuvette, incubated for 10–20 min, and the absorbance was measured again. The results were calculated using the Lambert–Beer equation. L-lactic acid concentrations were normalized based on the change in live cell count determined by crystal violet staining of the culture dish over time.

The obtained living cell counts were normalized by division with the protocol described in the previous section. The L-lactic acid/glucose ratios were used to assess the changes in the metabolic activities of the cells [30,31,32,36].

### 4.6. Statistical Analysis

The results are expressed as mean ± standard deviation (SD) from at least three independent experiments in all of the tests described above. One-way analysis of variance with Tukey’s test was conducted for statistical analysis [47].

## Data Availability

The original contributions presented in the study are included in the article, further inquiries can be directed to the corresponding author.
